# Household costs of hospitalized dengue illness in semi-rural Thailand

**DOI:** 10.1371/journal.pntd.0005961

**Published:** 2017-09-22

**Authors:** Yesim Tozan, Pitcha Ratanawong, Maquines Odhiambo Sewe, Annelies Wilder-Smith, Pattamaporn Kittayapong

**Affiliations:** 1 Institute of Public Health, Heidelberg University Medical School, Heidelberg, Germany; 2 College of Global Public Health, New York University, New York, NY, United States of America; 3 Division of Social Science, New York University Abu Dhabi, Abu Dhabi, United Arab Emirates; 4 Center of Excellence for Vectors and Vector-Borne Diseases and Department of Biology, Faculty of Science, Mahidol University, Bangkok, Thailand; 5 Epidemiology and Global Health, Department of Public Health and Clinical Medicine, Umeå University, Umeå, Sweden; 6 Lee Kong Chian School of Medicine, Nanyang Technological University, Singapore, Singapore; Institute for Disease Modeling, UNITED STATES

## Abstract

**Background:**

Dengue-related illness is a leading cause of hospitalization and death in Thailand and other Southeast Asian countries, imposing a major economic burden on households, health systems, and governments. This study aims to assess the economic impact of hospitalized dengue cases on households in Chachoengsao province in eastern Thailand.

**Methods:**

We conducted a prospective cost-of-illness study of hospitalized pediatric and adult dengue patients at three public hospitals. We examined all hospitalized dengue cases regardless of disease severity. Patients or their legal guardians were interviewed using a standard questionnaire to determine household-level medical and non-medical expenditures and income losses during the illness episode.

**Results:**

Between March and September 2015, we recruited a total of 224 hospitalized patients (<5 years, 4%; 5–14 years, 20%, 15–24 years, 36%, 25–34 years, 15%; 35–44 years, 10%; 45+ years, 12%), who were clinically diagnosed with dengue. The total cost of a hospitalized dengue case was higher for adult patients than pediatric patients, and was US$153.6 and US$166.3 for pediatric DF and DHF patients, respectively, and US$171.2 and US$226.1 for adult DF and DHF patients, respectively. The financial burden on households increased with the severity of dengue illness.

**Conclusions:**

Although 74% of the households reported that the patient received free medical care, hospitalized dengue illness cost approximately 19–23% of the monthly household income. These results indicated that dengue imposed a substantial financial burden on households in Thailand where a great majority of the population was covered by the Universal Coverage Scheme for health care.

## Author summary

Dengue, an arbovirus infection with an explosive epidemic potential, is a major public health problem in Thailand and other developing countries in subtropical and tropical regions. Dengue illness often leads to school and work absenteeism, medical and non-medical expenditures, and foregone income. A growing literature shows that these illness related costs pose a severe economic burden on households, health care systems, and governments. We conducted a prospective cost-of-illness study to assess the costs and impact of hospitalized pediatric and adult dengue cases on households in a highly endemic area in eastern Thailand. We found that the total cost of a hospitalized dengue case accounted for about 19–23% the monthly household income. Although direct medical costs were covered for a majority of hospitalized dengue patients by the Thai Universal Coverage Scheme for health care, direct non-medical and indirect costs were of great economic significance to households. These hidden costs of dengue illness are likely to increase given the shift in the mean age of dengue cases in Thailand and other endemic countries in the region. High household costs of dengue illness justify efforts to improve the coverage of preventive and control measures against dengue in endemic areas.

## Introduction

Dengue, an arbovirus infection with an explosive epidemic potential, is a major public health problem in many tropical and subtropical countries today [1]. The incidence of dengue has dramatically increased over the past decades, with the number of symptomatic dengue infections reported to be doubling every 10 years between 1990 and 2013 [2]. Dengue hemorrhagic fever (DHF), a severe and potentially life-threatening form of the disease, has also reported to be increasing steadily during this period, driving the increase in hospitalization rates for dengue, particularly in children [3–7]. Yet, an increasing number of studies have shown that there is substantial under-reporting of dengue cases to national surveillance systems, which prevents an accurate estimation of the disease burden of dengue in endemic countries [8]. Despite its limited effectiveness and high cost, vector control is the mainstay of dengue control and outbreak response in endemic areas [9–12]. The disease is expected to further expand its geographical range due to favorable conditions provided by rapidly growing high density urban areas along with socio-economic changes [13], increased worldwide travel and trade [14], and climate change [15]. A growing literature shows that dengue imposes an enormous socioeconomic burden on households, health care systems, and governments in endemic countries [16–18], particularly during outbreaks [19–22].

Reported as a public health problem since the 1950s, dengue causes frequent outbreaks in Thailand and is hyperendemic with all four distinct serotypes of the dengue virus in circulation for more than five decades [23]. Although dengue has traditionally affected children, there has been a shift in the mean age of dengue cases towards older age groups in Thailand and other dengue hyperendemic countries in Southeast Asia [24–28]. Most dengue cases now occur in individuals aged 5–24 years [29], which account for one third of the total population in Thailand, and the disease is more common in adolescents and young adults [30]. The incidence of DHF varies widely from year to year, exhibiting as much as a tenfold difference between years [26]. During the period 2000–2011, the incidence of DHF was higher in children aged 5–14 years than those aged 15 years or older [29]. While the case fatality rate of dengue has been declining steadily over the past decade, the highest rates are seen in children aged 0–4 years [29].

Frequent and severe illness can cause considerable social and economic disruption to households by requiring one or multiple visits to health care providers and hospitalization. Dengue illness often leads to school and work absenteeism, medical and non-medical expenditures, and foregone income. Illness related costs incurred by patients and household members constitute a severe economic burden for households, particularly in developing country settings. To accurately assess the overall economic burden of dengue, cost-of-illness data at the household level are, therefore, essential. Within the context of a European Union funded research project on dengue [31], we conducted a prospective hospital-based cost-of-illness study to assess the cost and impact of hospitalized dengue cases on households in a highly endemic area in eastern Thailand. Previous cost-of-illness studies in Thailand focused primarily on pediatric dengue patients (aged under 15 years) [32–36]. In view of the shift in the age distribution of dengue cases, we expanded the focus to cover adult dengue patients (aged 15 years and above).

## Material and methods

### Study design

We conducted a prospective, hospital-based cost-of-illness study in Chacheongsao province in eastern Thailand. Chachoengsao is a highly endemic area for dengue with a population of 700,902 in 2015 [37] and a surface area of 5,351 km^2^. The province is divided into 11 districts with 93 sub-districts, and has one province-level and nine district-level hospitals in total. Historically an agriculture-based province with rice paddies, fruit plantations, and livestock, it has become industrialized in recent years, transitioning from rural to semi-rural and semi-rural to semi-urban.

One provincial-level and two district-level public hospitals participated in the study. The study population included hospitalized pediatric (aged under 15 years) and adult (aged 15 years and above) patients who were clinically diagnosed with dengue. All patients or their legal guardians were invited to participate in the study and asked to sign an informed consent form. Patients who did not give consent were excluded from the study. The recruitment period was from March to September in 2015 and overlapped with the peak season of dengue illness.

### Research procedures

We adapted a patient questionnaire, which was successfully used in previous cost-of-illness studies in several dengue endemic countries [17]. It was translated into and back-translated from Thai by two researchers who were fluent in both languages, and the discrepancies were resolved through discussion. The questionnaire was piloted on 10 patients and validated before its administration. It collected information on the demographic and socio-economic characteristics of the patients and other household members, the characteristics of dengue illness episodes, work and school absenteeism, health care service utilization, household health care spending and coping strategies, care provided to the patients by household members, and household income loss due to the dengue illness episode.

### Data collection and management

Patients or their legal guardians were interviewed in-person after recovery from the illness by six experienced public health officers, and each officer received a half-day one-on-one training about the study protocol. The interviews took place at the hospital, the patient’s workplace or home, or any other place convenient for the patient. Each interview lasted about 30–45 minutes. Patients or their legal guardians were compensated for their time with a stipend in the amount of 200 Thai Baht (THB) (US$5.8). We followed up with 5–10 patients by phone because there was missing data or inconsistent information in the completed questionnaires. Data were entered into a Microsoft Access Database (2015, Microsoft Corp, Redmond, WA) and analyzed using SAS 9.4 (SAS Institute Inc., Cary, NC).

### Analytical framework

The unit of analysis was a dengue case, defined as a documented acute febrile illness with a clinical diagnosis of dengue at the time of hospital discharge. This study examined all hospitalized dengue cases regardless of disease severity.

Household expenditures on dengue include direct medical and non-medical costs and indirect costs incurred by the household during the dengue illness episode. Direct medical costs comprised all household out-of-pocket payments for medical services received by the patient prior to and during hospitalization. Direct non-medical costs included out-of-pocket payments for transportation, food and lodging for the patient and accompanying household members while seeking and receiving medical care for the illness episode. Indirect costs incurred by the household were assessed as the sum of lost paid work by the patient and other household members aged 15 years and above while caring for the patient during the dengue illness episode. We valued lost paid work as the higher of the reported income loss or the estimated income loss calculated conservatively as the product of the minimum daily wage (300 THB [38]) in Thailand times the number of reported workdays lost by the patient or other household members. The value of time forgone from leisure or other non-market activities was not included in the calculation of indirect costs. If reimbursements were paid to the household by health and/or income protection insurance, the amount reported was subtracted from the sum of the direct and indirect costs for that particular household to arrive at a total cost per case. We also reported on the number of school days lost by the patient and other household members due to dengue illness, as well as the total number of days household members cared for the patient during the illness episode. All costs were presented as mean (± standard deviation, SD) and expressed in 2015 US$ based on 34.2 THB to 1US$ currency exchange rate [39].

### Ethics statement

The protocol for this study was reviewed and approved by the Ethical Review Boards of Mahidol University, Heidelberg University, Chachoengsao Provincial Public Health Office, and Buddhasothorn Hospital. Signed informed consent was obtained from all patients or their legal guardians. Participant information, such as gender, age, clinical diagnostic status and contact information, was obtained from the hospital records. All the data collected through the cost of illness questionnaire and the hospital records were analyzed anonymously.

## Results

### Characteristics of the study population and dengue illness episodes

**Fig 1:**
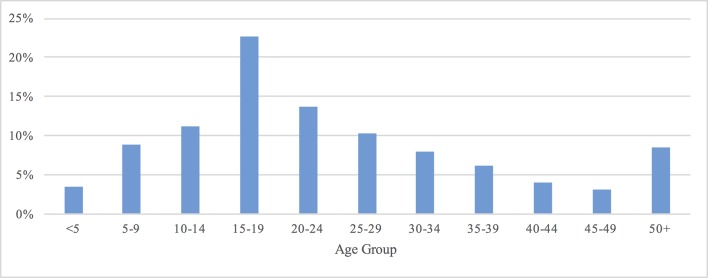
The age distribution of hospitalized dengue patients, Chacheongsao province, Thailand, 2015.

A total of 570 hospitalized patients, who were clinically diagnosed with dengue, were eligible to participate in the study. Of these hospitalized patients, 224 were recruited into the study. The general characteristics of hospitalized dengue patients and dengue illness episodes are summarized in Tables 1 and 2. Overall, 48% were female, and 24% were aged under 15 years of age. The age distribution of the study population is presented in Fig 1. The mean household size was 4.4 persons (SD 1.9). Among adult hospitalized patients, 18% had primary school education or less, 36% secondary school education, and 46% vocational/high school/college education or above. Of the 224 hospitalized patients, 168 (75%) and 56 (25%) had a clinical diagnosis of Dengue Fever (DF) and Dengue Hemorrhagic Fever (DHF), respectively, at the time of hospital discharge. About 73% of DF patients and 86% of DHF patients were aged 15 years or above. There were no deaths in this cohort of hospitalized dengue patients.

**Table 1:** General characteristics of the hospitalized dengue patients, Chacheongsao province, Thailand, 2015.

**Sex, n (%)**	
Male	117 (52)
Female	107 (48)
**Age distribution, n (%)**	
<15 years	53 (24)
≥15 years	171 (76)
**Highest level of education among adult patients, %**	
Primary school or less	18
Secondary school	34
Vocational school or more	45
Missing	3
**Highest level of education in household with pediatric patients, %**	
Primary school or less	27
Secondary school	18
Vocational/high school, college or more	49
Missing	6
**Patients currently studying, n (%)**	
53 patients < 15 years of age	48 (91)
171 patients ≥15 years of age	43 (25)
**Patients currently working, n (%)**	
53 patients <15 years of age	2 (4)
171 patients ≥15 year of age	97 (57)
**Household members currently studying, n (%)**	
602 household members	106 (17)
**Household members currently working, n (%)**	
602 household members	312 (52)

**Table 2:** General characteristics of the hospitalized dengue cases by patient age group and disease severity, Chacheongsao province, Thailand, 2015.

	Patient age group		
	<15 years	≥15 years	All
**Dengue diagnosis at hospital discharge, n (%)**			
DF	45 (85)	123 (72)	168 (75)
DHF	8 (15)	48 (28)	56 (25)
All	53 (100)	171 (100)	224 (100)
**Duration of illness (days), mean±SD**			
DF	8.0±2.1	9.5±2.5	9.0±2.5
DHF	10.0±2.9	10.5±4.8	10.5±4.6
All	8.3±2.3	9.9±3.6	9.5±3.4
**Patient feeling bad or very bad (days), mean±SD**			
DF	4.3±2.6	3.5±2.1	3.7±2.2
DHF	2.5±0.8	4.5±2.2	4.3±2.2
All	4.0±2.5	3.8±2.2	3.8±2.2
**Time between onset of illness and seeking first treatment, n (%)**			
<24 hours	25 (48)	61 (37)	86 (39)
24–48 hours	8 (15)	47 (28)	55 (25)
>48 hours	19 (37)	59 (35)	78 (36)
**Duration of hospital stay (nights), mean**±**SD**			
DF	4.0±2.6	3.7± 1.5	3.8±1.9
DHF	4.6±1.3	4.2±2.1	4.3±2.0
All	4.1±2.5	3.8±1.7	3.9±1.9

Overall, hospitalized dengue patients reported 9.5 days (SD 3.4) of illness, including 3.8 days (SD 2.2) during which the patient felt bad or very bad. The mean duration of illness was 8.0 days (SD 2.1) for pediatric DF patients and 10.0 days (SD 2.9) for pediatric DHF patients, including 4.3 days (SD 2.6) and 2.5 days (SD 0.8) during which the patient felt bad or very bad, respectively. Adult DF and DHF patients, respectively, reported 9.5 days (SD 2.5) and 10.5 days (SD 4.8) of illness, including 3.5 days (SD 2.1) and 4.5 days (SD 2.2) during which the patient felt bad or very bad.

### Utilization of health care services

Forty-seven percent of caretakers reported seeking care for their children within 24 hours of onset of illness, 15% reported seeking care one to two days after onset of illness, and 36% waited more than two days. Thirty-six percent adult patients sought care within 24 hours after onset of illness, 27% sought care one to two days after onset of illness, and the remaining 35% waited more than two days. Table 3 presents the type of health facility visited and the type of health provider consulted by hospitalized dengue patients during their illness episode. Fifty-four percent of the patients sought care at a hospital first and got hospitalized during their first visit, followed by 15% visiting a doctor’s office and 14% a pharmacy. Sixty-eight percent of the first visits occurred in a public health facility. About 51% and 17% of the patients reported a second and a third visit, respectively, where 64% and 84% of these visits resulted in hospitalization, and 82% and 97% of them occurred in public health facilities. Two patients had multiple hospitalizations during their illness episode.

**Table 3:** Type of health facilities visited and health providers consulted by hospitalized dengue patients, Chacheongsao province, Thailand, 2015.

	First visit	Second visit	Third visit	Fourth visit	Fifth visit
**Type of health facility, n (%)**					
Hospital	120 (54)	73 (64)	32 (84)	2 (50)	2 (100)
Emergency care	6 (3)	7 (6)	1 (3)	0 (0)	0 (0)
Outpatient department at a hospital	13 (6)	13 (11)	4 (10)	2 (50)	0 (0)
Health center	20 (9)	3 (3)	0 (0)	0 (0)	0 (0)
Doctor’s office	34 (15)	13 (11)	0 (0)	0 (0)	0 (0)
Laboratory	0 (0)	1 (1)	1 (3)	0 (0)	0 (0)
Pharmacy	31 (14)	4 (4)	0 (0)	0 (0)	0 (0)
**Type of health provider, n (%)**					
Public provider	152 (68)	93 (82)	37 (97)	4 (100)	2 (100)
Private provider	68 (30)	20 (18)	1 (3)	0 (0)	0 (0)
Don’t know	4 (2)	1 (0)	0 (0)	0 (0)	0 (0)

Dengue patients spent, on average, 3.9 (SD 1.9) nights in the hospital. While the mean number of hospital nights for pediatric DF and DHF patients was 4.0 (SD 2.6) and 4.6 (SD 1.3), respectively, adult DF and DHF patients spent, on average, 3.7 (SD 1.5) and 4.2 (SD 2.1) nights in the hospital, respectively. None of the hospitalized dengue patients reported receiving care in the intensive care unit.

Dengue illness affected school attendance and productive activities of the patients and other household members. Table 4 presents the mean number of school days missed and work days lost. Of the 91 hospitalized dengue patients who were studying at the time of illness, 79 reported missing school with an average of 6.8 (SD 4.0) days. Of the 53 pediatric dengue patients, 48 were in school at the time of illness, and 45 missed an average of 6.5 (SD 3.8) days of school. The mean number of school days missed was 6.6 (SD 3.9) and 6.1 (SD 3.1) days for pediatric DF and DHF patients, respectively. Of the 171 hospitalized adult patients, 43 were in school at the time of illness, and 34 reported missing school with an average of 7.2 (SD 4.2) days. Of the 99 hospitalized dengue patients who were working for pay at the time of illness, 97 were adult patients, and 94 lost an average of 6.9 (SD 3.5) days of work due to the illness episode. The mean number of work days lost for adult DF and DHF patients was 6.6 (SD 3.6) and 7.6 (SD 3.1) days, respectively. The burden of a hospitalized dengue case on household members was also considerable. Of the 602 household members, 52% and 17% reported to be working and attending school, respectively. On average, household members missed 1.2 (SD 2.9) days of school and lost 4.1 (SD 3.9) days of work. The mean total number of days cared for the patient during the illness episode was 7.2 (SD 4.9) per household.

**Table 4:** Impact of a hospitalized dengue case on patients’ school attendance and productive activities, Chacheongsao province, Thailand, 2015.

	Pediatric patients (<15 years)	Adult patients (≥15 years)	All
**Days of school missed, mean±SD**			
DF	6.6±3.9	5.9±2.7	6.3±3.5
DHF	6.1±3.1	10.3±5.7	8.6±5.1
All	6.5±3.8	7.2±4.2	6.8±4.0
**Days of work lost, mean±SD**			
DF	7.0±2.8	6.6±3.6	6.6±3.6
DHF	-	7.6±3.1	7.6±3.1
All	7.0±2.8	6.9±3.5	6.9±3.5

### Household costs of a hospitalized dengue case

Table 5 presents the direct, indirect and total costs of hospitalized dengue cases to households by patient age category and disease severity. The mean total household cost of a hospitalized pediatric and adult dengue case was US$155.4 (SD 112.1) and US$186.8 (SD 184.7), including a mean reimbursement of US$7.7 (SD 24.1) and US$20 (SD 138.9), respectively. The direct costs for pediatric and adult patients amounted to US$81.9 (SD 76.5) and US$109.3 (SD 190.4), constituting 52% and 59% of the total household costs while the mean indirect costs were US$81.1 (SD 66.3) and US$97.5 (SD 110.3), respectively. The direct non-medical costs accounted for the majority of the direct costs to households regardless of patient age category, and were US$67.2 (SD 66.4) and US$78.6 (SD 94.7), constituting 82% and 72% of the direct costs, respectively, the rest being the direct medical costs.

**Table 5:** Household costs of hospitalized dengue illness by patient age group and disease severity, Chacheongsao province, Thailand, 2015. All costs are reported in 2015 US$.

	DF	DHF	All
**Pediatric patients (<15 years), mean±SD**			
Direct cost	80.0±76.3	94.1±82.6	81.9±76.5
* Medical cost*	*15*.*8*±*25*.*7*	*8*.*3*±*10*.*7*	*14*.*7*±24.3
* Non-medical cost*	*64*.*2*±*63*.*8*	*85*.*9*±*84*.*4*	*67*.*2*±66.4
Indirect cost	81.7±68.7	77.7±53.5	81.1±66.3
Total cost	153.6±115.3	166.3±96.3	155.4±112.1
**Adult patients (≥ 15 years), mean±SD**			
Direct cost	96.5±127.1	141.6±294.6	109.3±190.4
* Medical cost*	20.0±52.0	57.6±246.8	*30*.*7*±*138*.*8*
* Non-medical cost*	76.5±99.8	84.0±81.3	*78*.*6*±*94*.*7*
Indirect cost	84.7±80.1	129.8±160.2	97.5±110.3
Total cost	171.2±167.2	226.1±220.1	186.8±184.7

Overall, the total household cost of a hospitalized dengue case increased with disease severity. The mean total cost of a pediatric DF and DHF case to households was US$153.6 (SD 115.3) and US$166.3 (SD 96.3), respectively. Adult patients reported a mean household cost of US$171.2 (SD 167.2) and US$226.1 (SD 220.1) for a DF and DHF case, respectively. The direct non-medical costs similarly accounted for the majority of the direct costs to households regardless of dengue disease severity. Among adult hospitalized patients, the direct medical costs and the indirect costs were notably higher for DHF cases compared to DF cases.

Overall, 74% of the households reported that the patient received free medical care during their illness episode, and 62% reported that the patient was covered by the Thai Universal Coverage Scheme for health care. A great majority of the households (94%) reported not borrowing money from outside the household or selling or transfering any household assets to finance the dengue illness episode. About 27% reported using household savings, and 41% reported that other household members helped finance the dengue illness episode.

## Discussion

This was a prospective cost-of-illness study, which aimed to quantify the direct, indirect and total costs of hospitalized dengue cases to patients and their households in a highly endemic, semi-rural area in eastern Thailand. Overall, wide regional variations in dengue incidence occur annually in Thailand [29]. During the period from 2014 to 2016, dengue-related morbidity and mortality rates in Eastern region where Chacheongsao province is located were notably higher than the national rates [40]. In the study year of 2015, dengue morbidity and mortality rates were 328.7 and 0.6 per 100,000 population for the Eastern region, compared to the national rates of 222.6 and 0.23 per 100,000 population, respectively [41]. While most published cost-of-illness studies on dengue have focused on pediatric patients, this study included adult patients in view of the recent shift in the mean age of dengue cases reported in Thailand and other endemic countries in Southeast Asia.

The average household cost of a hospitalized dengue case in Chachoengsao province was US$153.6–166.3 and US$171.2–226.1 for pediatric and adult patients, respectively. These costs fell within the range of those reported in other studies for Thailand, ranging from US$44 in 2001 [42] to US$118 in 1994 [43], and for adult patients from US$138 to US$162 in 1994 (unadjusted costs) [43]. Overall, the total cost of a hospitalized dengue case to the household was higher for adult patients than pediatric patients. Unsurprisingly, the severity of dengue illness was found to increase the financial burden on households due to more prolonged and complicated treatment and longer illness period.

The direct medical costs constituted only a small portion of the direct costs and the total costs, and were US$14.7 for pediatric patients and US$30.7 for adult patients. This could be explained by the fact that the majority of the study population was covered by the Thai Universal Coverage Scheme for health care and paid THB30 (US$ 0.88) per visit or admission. This co-pay was waived for children and elderly and for households with income less than THB2,800 (US$81.9) per month. The direct non-medical costs increased with the severity of dengue illness for both pediatric and adult patients. Our findings showed that the indirect costs were as significant as the direct costs, constituting about half of the total costs to the households. The indirect costs reported for pediatric patients in this study were similar to those reported by other studies, ranging from US$20 in Khamphaeng Phet in 2001 to US$42 and US$51 for clinically diagnosed and laboratory confirmed dengue, respectively, in Khon Kaen in 2005 (unadjusted costs) [44].

The average monthly household income in Chacheongsao province was THB27,555 (US$806) in 2015 [45]. Our findings showed that a hospitalized dengue episode cost approximately 19–23% of the monthly household income, which was lower than what was previously reported for Thailand as 37% [42]. However, some households had to use additional financial sources, such as household savings (27%), or sought financial assistance from other household members (41%) to finance the dengue illness episode. These coping strategies that deal with direct costs of illness can potentially undermine future income streams and threaten the economic sustainability of households. Our study potentially underestimated the total costs of dengue illness to households because dengue episodes tend to cluster at the household level, affecting multiple household members simultaneously. An earlier study in Thailand found that dengue affected an average of 1.4 family members per household per episode [42].

There were several limitations to this study. It was conducted in three hospitals in a single province with a focus on hospitalized clinically diagnosed dengue cases in the public health sector. Previous cost of illness studies did not find any significant differences in health seeking behavior and overall costs between laboratory confirmed and clinically diagnosed dengue cases, and laboratory confirmed dengue and non-dengue febrile cases [34,46]. The total cost of a hospitalized dengue case to a household is, however, expected to vary depending on the type of hospital where care is received and whether the hospital is public or private. Similar to other cost of illness studies, this study relied on self-reported costs, which are prone to recall bias. It has been shown that a recall timeframe of one year is appropriate for rarely used health care services, such as hospitalization, and much shorter timeframes are recommended for more frequently used services, such as doctor visits [47]. To minimize potential recall bias, patients were contacted within one to six weeks of hospital discharge to check up on patient recovery progress and set up an interview date, and were interviewed about within four to ten weeks of recovery. The issue of self-reported costs is particularly important when considering the indirect costs associated with an illness episode. The method of asking patients how many days they could not work has been shown to overestimate the productivity losses from a disease [48]. This is mainly because patients, particularly in low and middle income countries, would not have been working for all of those days in the absence of a disease. It is also a common coping strategy that other household members fill in for a sick person or for a parent caring for a sick child to sustain the household productivity [48]. We, therefore, estimated the value of lost paid work conservatively using Thailand’s minimum daily wage when the patient or household member reported number of work days lost rather than income loss. Despite these limitations, our study has the most comprehensive cost-of-illness data for hospitalized dengue cases in Thailand to date.

The costs of inpatient care services absorbed by the government through the Thai Universal Coverage scheme is beyond the scope of this study. We, however, received the hospital bills of 89 patients from the provincial hospital. The hospital bills indicated the length of hospital stay and the service fees for inpatient care provided during hospitalization for each dengue patient. The mean inpatient service fees for DF and DHF cases in the provincial hospital were US$145.7 (SD 78.9) and US$152.1 (SD 107.1) for pediatric patients and US$132.2 (SD 62.5) and US$144.9 (SD 82.3) for adult patients, respectively. The service fees are negotiated fees paid to hospitals by the public health insurance. Therefore, they do not cover the full economic cost of inpatient care provided at hospitals and are not comparable to the direct medical costs of hospitalized dengue cases reported in other costing studies. Still, these service fees are relatively high compared to the estimated direct medical costs of hospitalized DF and DHF cases at secondary care hospitals in Colombo district, Sri Lanka: US$ 51 (SD 1) and US$129 (SD 3) for pediatric patients and US$32 (SD 1) and US$91 (SD 2) for adult patients (2012 US$), respectively [22].

### Conclusions

This study showed that dengue related illness imposes financial hardship on households in Thailand when hospitalization is required. Although direct medical costs were covered for a majority of hospitalized patients by the Thai Universal Coverage Scheme for health care, direct non-medical and indirect costs were of great economic significance to households. These hidden costs of dengue illness are likely to increase given the shift in the mean age and severity of dengue cases in Thailand and other dengue affected countries in the region. This begets the question of whether households can be protected from these hidden costs through innovative policy measures in dengue endemic countries. To fully understand the economic impact of dengue illness on households, it is necessary to collect cost of illness data for both hospitalized and non-hospitalized dengue cases and in both the public and private health sectors. The total cost of a hospitalized dengue case in public facilities accounted for about 19–23% the monthly household income. High household costs of dengue illness strongly justify efforts to improve the coverage of preventive and control measures against dengue. Such cost of illness data are also key to evaluating the cost-effectiveness of these measures, including dengue vaccines.
